# Security and Privacy Techniques in IoT Environment

**DOI:** 10.3390/s21010001

**Published:** 2020-12-22

**Authors:** Jerry Chun-Wei Lin, Kuo-Hui Yeh

**Affiliations:** 1 Department of Computer Science, Electrical Engineering and Mathematical Sciences, Western Norway University of Applied Sciences, Inndalsveien 28, 5063 Bergen, Norway; 2 Department of Information Management, National Dong Hwa University, No. 1, Sec. 2, Da Hsueh Rd., Shoufeng, Hualien 97401, Taiwan; khyeh@gms.ndhu.edu.tw

Due to rapid technical advancements, many devices in the Internet of Things (IoT) environment, such as embedded systems, mobile devices, actuators, and sensors (all of which can be referred to as smart things), can receive huge amounts of information through data exchanging and interconnection. In this context, it is important to preserve individual privacy and secure shared data. Thus, privacy and security has attracted a great deal of attention and research interest in recent decades. Hundreds of security solutions have recently been discussed for the IoT environment. Since many criteria and parameters must be considered with regard to privacy and security issues, it is critical to discuss and develop new methodologies and techniques by adopting evolutionary computations. The objective of this Special Issue on “Security and Privacy Techniques in IoT Environments” was to compile recent research efforts dedicated to studying and developing security and privacy issues related to IoT devices and the IoT environment. The Special Issue solicited high quality, unpublished work on recent advances in new methodologies for security and privacy solutions, as well as theories and technologies proposed to defend IoT-oriented applications against adversarial or malicious attacks. The number of selected/accepted papers for publication was 15, and their main contributions are described below.

The first paper [1] proposes a new intrusion detection improved conditional variational AutoEncoder with a deep neural network (ICVAE-DNN) model. The ICVAE part is utilized to learn and explore potential sparse representations between network data features and classes. After that, the decoder of the ICVAE generates new attack samples according to the specified intrusion categories to balance the training data and increase the diversity of training samples. This approach improves the detection rate of imbalanced attacks. Using on the trained ICAVE encoder, one can automatically reduce data dimensions and initialize the weight of DNN hidden layers. Thus, the DNN part can easily achieve global optimization through back propagation and fine tuning. The results of this research showed that the developed ICVAE-DNN achieved better performance compared to three well-known oversampling methods in data augmentation, and it also outperformed six well-known models in detection performance. Furthermore, the ICVAE-DNN showed a better overall accuracy, detection rate, and false positive rate than nine state-of-the-art intrusion detection methods.

The second paper [2] proposes a Context-Aware Trustworthy Social Web of Things System (CATSWoTS) that addresses the interoperability issue by incorporating web technologies. The aspect of social web in the designed system helps get recommendations from social relations. It first states the importantness of context dependency of trust and quality of service (QoS) criteria to identify and recommend trustworthy Web of Things (WoT) applications. Thus, the parameters of context awareness and the constraints of QoS are considered in the designed system. The CATSWoTS evaluates service providers based on the mentioned parameters and constraints, and then it identifies a suitable service provider by using a rule-based collaborative filtering approach. Experiments were conducted using a real QoS dataset to evaluate the performance of the designed CATSWoTS. In summary, the designed CATSWoTS performed well by dynamically identifying and recommending trustworthy services, as per the requirements of a service seeker.

The third paper [3] analyzed a recently proposed authentication protocol for Wireless Sensor Networks (WSNs) proposes its enhancement to overcome the shortcomings that were identified during the analysis. The analyzed protocol is suitable for resource-constrained sensor nodes due to the use of lightweight cryptographic primitives such as hash functions and symmetric encryption. The protocol uses user’s biometrics to ensure user-privacy, which make this protocol a three-factor authentication protocol. However, the protocol imposes a heavy computational load over the gateway node in the pursuit of providing user-anonymity, thereby opening the doors for denial of service (DoS) attacks. Moreover, if an adversary captures a sensor node, then the adversary can use the information gained from the captured sensor node to impersonate a legal user to the gateway node. Additionally, the adversary can impersonate the other sensor nodes to deceive the user and can decrypt all the cipher-texts of the user. The designed framework thus solves the previous limitation regarding authentication protocol for Wireless Sensor Networks (WSNs).

The fourth paper [4] proposes a privacy-preserving broker— Attribute-Based Encryption (ABE) for IoT—and makes the IoT gateway the broker. In general, the broker is more powerful than other devices. Thus, the ABE scheme is separated into two parts: the policy embedding task and the encryption task. The costly job, which is the policy embedding task, is moved to the broker, while the encryption task is kept in the sensor to stop the broker from eavesdropping. Therefore, the scheme takes care of security and practicality at the same time. Additionally, this paper points out an important issue about data privacy in the cloud. Since data are processed there, the cloud may be forced to open data to third parties. Traditional encryption schemes are not able to protect user privacy in this attack model. This paper makes the proposed scheme a non-commitment encryption scheme. Thus, the cloud can deal with outside coercion by providing fake data. To summarize, this paper designed an overall cloud-assisted IoT system, including field devices, IoT gateways, and cloud services, to maintain data privacy.

The fifth paper [5] proposes an intrusion detection method based on a deep belief network (DBN) optimized by particle swarm optimization (PSO). First, a classification model based on the DBN was constructed, and then the PSO algorithm was used to optimize the number of hidden layer nodes of the DBN to obtain an optimal DBN structure. Simulations were conducted on a benchmark intrusion dataset, and the results showed that the accuracy of the DBN-PSO algorithm was 92.44%, which was higher than those of a support vector machine (SVM), artificial neural network (ANN), deep neural network (DNN), and AdaBoost. It could be seen from comparative experiments that the optimization effect of PSO was better than those of a genetic algorithm, a simulated annealing algorithm, and a Bayesian optimization algorithm. The method of PSO-DBN provides an effective solution to the problem of the intrusion detection of Unmanned Aerial Vehicles (UAV) networks. 

The sixth paper [6] proposes a Blockchain-based Integrated Network Function Management (BINFM) scheme where the Network Address Translation (NAT), mobility, and security management are handled simultaneously. The proposed scheme is advantageous because by using blockchain and a query/reply mechanism, each peer can easily obtain the necessary parameters required to handle the NAT, mobility, and security management in a batch. In addition, this paper explains how the proposed scheme guarantees secure end-to-end data transfers with the use of one time session keys. Finally, it is proved that, compared to the existing vertical model, the proposed scheme improves performance on latency from the viewpoints of mobility and security. 

The seventh paper [7] proposes the building of a semi-lattice of mined high-utility itemsets. Additionally, the designed model generates non-redundant high utility association rules from the semi-lattice structure and applies the definition of non-redundant high utility association rules to generate a more accurate set of results. The new algorithm was validated on popular data mining datasets with both sparse and dense types to prove its faster runtime and better memory consumption compared to previous research in terms of performance and data correctness. Though this paper is not relevant to the security issue in the Special Issue, this new algorithm can be integrated with variety of applications and can be combined well with external systems, e.g., Internet of Things and distributed computer systems, in order to help management visualize customer needs, as well as make more efficient business strategies. The approach also brings some new open research directions for the future, e.g., applying non-redundant high utility association rules as representative training sets for machine learning algorithms or systems to speed-up decision-making activities or predict the trends of customers.

The eighth paper [8] aimed to ensure a secure IoT environment by proposing an efficient key management technique that uses a combination of symmetric and asymmetric cryptosystems. Their proposal considers a set of smart objects (SOs) that is capable for registering, generating, and distributing keys in the transmission of IoT data. They use the open source Message Queuing Telemetry Transport (MQTT) protocol to facilitate communications between source and destination nodes. The suitability of the proposed approach was measured experimentally, and the results were comparable to existing work with respect to key conversion time, algorithm execution time, the number of reuse connections, and bandwidth utilization. 

The ninth paper [9] focuses on the study of mimicry from the standpoint of an uncharted terrain: masquerade detection based on analyzing locality traits. Its main contributions are as follows: (1) it reviews the evasion of masquerader detection systems based on an analysis of locality traits, (2) it introduces two novel evasion tactics (locality-based mimicry by action pruning and locality-based mimicry by noise generation) that show the weaknesses of the conventional machine learning-based solutions applied to masquerade detection, (3) it provides experimental evidence of the vulnerability of state-of-the-art classifiers against those threats, and (4) it presents a comprehensive discussion of the empirical results and research findings. 

The tenth paper [10] introduces an effective sequential convex estimation optimization (SCEO) algorithm to keep the security of the physical layer in a wireless communication network with three nodes. The experimental results showed that using the SCEO algorithm resulted in a maximized performance and advanced convergence in the transmission. While considering likely security issues when there is an active multiple eavesdropper in a network, the authors broadened their research to the development of a fast privacy rate optimization algorithm for a multiple-input, multiple-output, multiple-eavesdropper (MINOME) scenario since it could be applied to 5G and IoT security. The investigation results illustrated that the algorithm could significantly achieve good performance without optimizing parameters. The authors also made use of a rate constraint and the self-interference of a full-duplex transmission at the receptive node, which makes the proposed technique’s performance unique when compared to techniques proposed in previous studies.

The authors of the eleventh paper [11] carried out an in-depth investigation of state-of-the-art systems and created a taxonomy that describes, characterizes, and highlights the main limitations of existing solutions. The proposed taxonomy provides a comprehensive overview of the reasons for the deployment of the solutions and the scenarios in which they operate. The results of this study demonstrated the main benefits and drawbacks of each solution set when applied to specific scenarios by examining current trends and future perspectives, e.g., the adoption of emerging technologies based on Cloud and edge (or fog) computing.

The twelfth paper [12] proposes an IoT-friendly subset representation called combinatorial subset difference (CSD) that generalizes the existing subset difference (SD) method by allowing for wildcards (*) in any position of a bitstring. Based on the CSD representation, the authors first propose an algorithm to construct a CSD subset and a CSD-based public key broadcast encryption scheme. By providing the most general subset representation, the proposed CSD-based construction achieves a more minimal header size compared the existing broadcast encryption. The experimental results showed that the proposed CSD could save header size by 17% on average and more than 1000 times when assuming a specific IoT example of an Internet Protocol (IP) address with 20 wildcards and 220 total users compared to SD-based broadcast encryption. The authors proved the semantic security of CSD-based broadcast encryption under the standard l-Bilinear Diffie-Hellman Exponent (l-BDHE) assumption, and they extended the construction to a chosen-ciphertext-attack (CCA)-secure version.

The thirteenth paper [13] first overviews the shortcomings of the existing two privacy protection architectures and privacy protection technologies. Then, the authors propose a location privacy protection method based on blockchain. The proposed decentralized location privacy protected architecture is used to protect user’s location privacy in Location-Based Services (LBS). The framework uses multiple private blockchain networks to decentralize user transaction records, thereby enabling the system to achieve decentralization. The proposed method satisfies the principle of k-anonymity privacy protection and does not need the help of trusted third-party anonymizing servers. The combination of multiple private blockchains can disperse the user’s transaction records, which can provide users with stronger location privacy protection without reducing the quality of service. The authors also propose a reward mechanism to encourage user participation. Finally, the authors implemented the proposed approach in the Remix blockchain to show its efficiency, which further indicated the potential application prospect for the distributed network environment. The authors were committed to solving the problem of privacy leakage when people use the IoT to obtain services, which is exactly related to this Special Issue.

The fourteenth paper [14] proposes a novel mutual two-factor authentication protocol between a server and an IoT device that only requires the use of hash functions. Two-factor authentication presents a strong security approach against cyberattacks. The authors also provide a mechanism for secure session key establishment. Furthermore, the concept of the proposed protocol in terms of cyberattack assessment, formal security analysis, and computational complexity is presented. A careful analysis validated the correctness of the proposed authentication protocol, and the proposed cyber defense assessment showed that it is impossible to compromise the protocol even if an adversary conducts an invasive attack against the IoT device. The proposed authentication mechanism is the first mechanism that replaces the traditional use of encryption for authentication with Hash-based Message Authentication Code (HMAC) computation. It deploys two Physical Unclonable Functions (PUFs) in the IoT device to offer the desired level of security. It also achieves the required low computational complexity for power-constrained IoT applications.

The fifteenth paper [15] summarizes the effort at identifying and evaluating publicly available sources of information about vulnerabilities, focusing on their usefulness in the scope of the IoT. The results of the proposed search showed that there is not yet a single satisfactory source covering the vulnerabilities affecting IoT devices. Most sources have only focused on software vulnerabilities. Some services covering the IoT already exist, but they are currently not receiving updates, and some of them are currently defunct and no longer available on the internet. Finally, the authors found a few sources dedicated to the industrial control system (ICS). The identification of the data sources is the first step on the road to create a publicly-available IoT vulnerability database that will improve the security of the IoT environment in a similar way to how national vulnerability databases help secure computing systems.
